# Melatonin Inhibits Annulus Fibrosus Cell Senescence through Regulating the ROS/NF-*κ*B Pathway in an Inflammatory Environment

**DOI:** 10.1155/2021/3456321

**Published:** 2021-08-18

**Authors:** Jing Li, Jianghua Li, Chengzhang Cao, Jianhua Sun, Sibo Wang, Zhi Ruan, Lei Chen, Ke Li

**Affiliations:** ^1^Orthopaedic Center, The First Affiliated Hospital of Shihezi University Medical College, Shihezi, Xinjiang 832008, China; ^2^Department of Preventive Medicine, School of Medicine, Shihezi University, Shihezi, Xinjiang 832008, China

## Abstract

Inflammation response is an important reason for disc cell senescence during disc degeneration. Recently, melatonin is suggested to protect against disc degeneration. However, the effects of melatonin on annulus fibrosus (AF) cell senescence are not fully studied. The main purpose of this study was to investigate the effects of melatonin on AF cell senescence in an inflammatory environment and the underlying mechanism. Rat disc AF cells were cultured in a medium with tumor necrosis factor-*α* (TNF-*α*). Melatonin was added along with the medium to observe its protective effects. Compared with the control AF cells, TNF-*α* significantly declined cell proliferation potency and telomerase activity, elevated senescence-associated *β*-galactosidase (SA-*β*-Gal) activity, upregulated protein expression of senescence markers (p16 and p53), and increased reactive oxygen species (ROS) content and activity of the NF-*κ*B pathway. However, when the TNF-*α*-treated AF cells were incubated with melatonin, ROS content and activity of the NF-*κ*B pathway were decreased, and those parameters reflecting cell senescence indicated that AF cell senescence was also partly alleviated. Together, melatonin suppresses AF cell senescence through regulating the ROS/NF-*κ*B pathway in an inflammatory environment. This study sheds a new light that melatonin may be promising to retard inflammation-caused disc degeneration.

## 1. Introduction

Intervertebral disc degeneration- (IDD-) caused spine degenerative diseases are very common in orthopedic outpatients. It is reported that approximately 80% of adults suffer IDD-induced low back pain for at least once in a lifetime [1]. Because of its high morbidity, IDD brings a heavy socioeconomic burden to the social heath care system in each country. Currently, lots of researchers are engaging in investigating the pathogenesis of IDD and seeking the potential approaches to biologically regenerate the degenerative disc tissues.

Enhanced inflammation response is identified in degenerative disc tissues by lots of scholars and has been proven to be associated with alterations in disc cell behaviors (i.e., cell viability and matrix metabolism) during disc degeneration [2, 3]. Hence, suppressing inflammation reaction-induced alterations in disc AF cell behaviors may be effective in retarding or alleviating disc degeneration.

Cell senescence is a newly identified cellular pathological feature within the degenerative disc tissues, and it plays an important role in disc degeneration [4–14]. Cell senescence also plays an important role in some other diseases, such as osteoarthritis, neurodegeneration, and renal diseases [15–18]. Once the active cells are senescent due to certain risk factors, they become inactive in proliferation and matrix biosynthesis [4, 9, 13, 19]. Previously, several studies have reported that inflammatory cytokines can accelerate disc cell senescence [8, 14, 20]. Annulus fibrosus (AF) tissue is the peripheral laminar structure of the intact intervertebral disc [21]. During disc degeneration, the integrity of AF is destroyed, which is reflected by tears and fissures within the disc AF tissue [22, 23]. Because the extracellular matrix secreted by disc cells is important for maintaining tissue structural integrity, inhibiting or suppressing AF cell senescence induced by inflammatory response may be promising for biologically retarding disc AF degeneration.

Melatonin (N-acetyl-5-methoxytraptamine) is an indolyl hormone released from the pineal gland, which has protective effects on the nervous system and other organs through inhibiting oxidative damage [24, 25]. Inflammatory response often induces cellular oxidative stress reaction in disc cells [8, 14, 26, 27]. In this study, we mainly aimed to investigate whether melatonin can attenuate disc AF cell senescence in an inflammatory environment and the effects of oxidative stress reaction in this process.

## 2. Materials and Methods

### 2.1. Isolation and Culture of Disc AF Cells

All of the animal experiments were authorized by the Animal Care and Use Committee of the First Affiliated Hospital of Shihezi University Medical College (wydw2014-0129). All animal work was completed at the animal research center of the First Affiliated Hospital of Shihezi University Medical College. In total, thirty-six Sprague-Dawley rats were used in this study. All rats were euthanized by excessive carbon dioxide inhalation. Briefly, after the thoracolumbar spine (L5-T10) discs were separated, the adjacent connective tissues were removed. Thus, the AF tissues were obtained under a surgical anatomy microscope and cut into small pieces (approximately 1 mm × 1 mm × 1 mm). By respective digestion with 0.25% trypsin (Gibco, USA) and 0.2% type I collagenase (Gibco, USA), the AF cell pellets were obtained after centrifugation (4°C, 1000 r/min, 5 minutes). Finally, the isolated AF cells were cultured or subcultured in DMEM/F12 medium supplemented with 15% fetal bovine serum (FBS, Gibco, USA) in an incubator containing 5% CO_2_ at 37°C. The passage 2 AF cells were used in this study. AF cells in the control group were still cultured in the baseline culture medium. AF cells in the experiment group were incubated by the baseline culture medium with TNF-*α* (50 ng/mL). To investigate the potential protective role of melatonin, it was added into the culture medium of the experimental group (1.0 mM, which is referred to in a previous study [28]). After the control AF cells and experimental AF cells were cultured for 5 days with different test compounds, they were collected to perform the designed assays.

### 2.2. Cell Proliferation Assay

A CCK-8 Assay Kit (Beyotime, China) was used to evaluate AF cell proliferation. Briefly, AF cells were seeded onto the 6-well cell culture plate (3 × 10^3^ cells/well) and treated with different test compounds. On days 3 and 5, cultured medium was refreshed by baseline culture medium supplemented with the CCK-8 solution (1 : 10). After incubation for 30 minutes at 37°C in an incubator, the absorbance value at a wavelength of 450 nm was detected to reflect AF cell proliferation potency.

### 2.3. Senescence-Associated *β*-Galactosidase (SA-*β*-Gal) Staining

A SA-*β*-Gal Staining Kit (Beyotime, China) was used to evaluate SA-*β*-Gal activity on day 3. After culture, AF cells were washed with phosphate buffer solution (PBS) for one time and then fixed by 1 mL SA-*β*-Gal fixation solution for 15 minutes at room temperature. Thereafter, the fixation solution was removed, and AF cells were washed with PBS for 3 times again (3 minutes per time). Finally, AF cells were stained by staining solution overnight in an incubator at 37°C without CO_2_ exposure. The ratio of positive-stained AF cells was used to reflect SA-*β*-Gal activity.

### 2.4. Telomerase Activity Measurement

A commercial telomerase (TE) enzyme-linked immunosorbent assay (ELISA) kit (Mlbio, China) was used to measure telomerase activity on day 5. After culture, AF cells were treated with RIPA lysis buffer (Beyotime, China) for 15 minutes in an ice box. Then, the absorbance value at 450 nm of a reaction system including the protein supernatant and the reaction buffer was measured. Finally, telomerase activity (IU/L) of AF cells was calculated according to the standard curve created using the standard and the absorbance value at 450 nm.

### 2.5. Reactive Oxygen Species (ROS) Detection Assay

The total intracellular ROS content was analyzed using a reactive oxygen species assay kit (Nanjing Jiancheng Bioengineering Institute, China). After being cultured for 5 days, AF cells were incubated with the DCFH-DA probe (10 *μ*M) for 20 minutes at 37°C in an incubator. Then, AF cells were further washed with the DMEM/F12 medium to remove the unbounded DCFH-DA probe. Finally, relative fluorescence units (RFU, excitation/emission wavelength: 490/585 nm) were detected to reflect the ROS content of AF cells.

### 2.6. Western Blot Assay

After culture, AF cells were lysed using the RIPA lysis buffer (Beyotime, China), and the total protein was extracted according to the manufacturer's instructions. Protein samples were separated by sodium dodecyl sulfate-polyacrylamide gel electrophoresis (SDS-PAGE) and transferred to the PVDF membrane. Then, PVDF membranes were sequentially incubated with primary antibodies (GAPDH: Abcam, ab181602; p16: Novus, NBP2-37740; p53: Abcam, ab26; NF-*κ*B: Abcam, ab16502; phospho-NF-*κ*B: Abcam, ab86299; all diluted at 1 : 2000) and secondary antibodies according to the standard procedure. After protein bands were developed using ECL Plus (Amersham Pharmacia Biotech, Umea, Sweden), densitometry analysis was finished using the ImageJ software (National Institutes of Health, USA).

### 2.7. Statistical Analysis

Each test was performed independently for three times. All data are expressed as the means ± SD. Statistical significance was analyzed by the one-way analysis of variance (ANOVA) using SPSS software (Version 18.0), followed by LSD test for comparison between each two groups. A statistical significance was indicated when *p* < 0.05.

## 3. Results

### 3.1. Cell Proliferation

**Figure 1:**
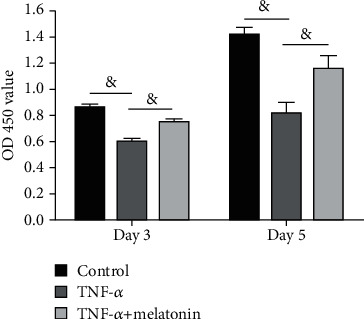
Analysis of annulus fibrosus cell proliferation. Annulus fibrosus (AF) cell proliferation potency was evaluated by the CCK-8 assay. All data are expressed as mean ± SD (*n* = 3). ^&^Indicates a statistical difference (*p* < 0.05).

Compared with the control group, the OD 450 value of the TNF-*α* group was significantly decreased, whereas addition of melatonin into the TNF-*α* group partly increased the OD 450 value (Figure 1).

### 3.2. SA-*β*-Gal Staining

**Figure 2:**
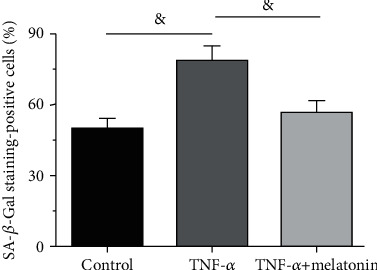
Analysis of senescence-associated *β*-galactosidase (SA-*β*-Gal) staining-positive rate. SA-*β*-Gal activity of annulus fibrosus (AF) cells was measured using a senescence *β*-galactosidase staining kit. All data are expressed as mean ± SD (*n* = 3). ^&^Indicates a statistical difference (*p* < 0.05).

Results showed that the ratio of SA-*β*-Gal staining-positive AF cells in the TNF-*α* group was significantly increased compared with that in the control cells. However, addition of melatonin partly decreased the ratio of SA-*β*-Gal staining-positive AF cells in the TNF-*α* group (Figure 2).

### 3.3. Telomerase Activity

**Figure 3:**
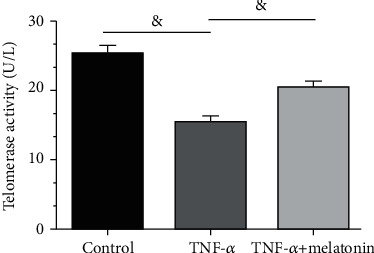
Analysis of telomerase activity. Telomerase activity of annulus fibrosus (AF) cells was evaluated using a chemical kit. All data are expressed as mean ± SD (*n* = 3). ^&^Indicates a statistical difference (*p* < 0.05).

Compared with the control cells, the TNF-*α* group showed a significantly decreased telomerase activity, whereas addition of melatonin into the TNF-*α* group partly increased the telomerase activity (Figure 3).

### 3.4. ROS Content

**Figure 4:**
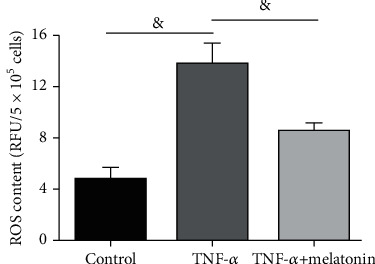
Analysis of intracellular reactive oxygen species (ROS) content. The intracellular ROS content in annulus fibrosus (AF) cells was measured by the fluorescent probe DCFH-DA. All data are expressed as mean ± SD (*n* = 3). ^&^Indicates a statistical difference (*p* < 0.05).

Compared with the control cells, the total intracellular ROS content in the TNF-*α* group was significantly increased, whereas melatonin partly declined the intracellular ROS content of AF cells in the TNF-*α* group (Figure 4).

### 3.5. Protein Expression of Senescence Markers

**Figure 5:**
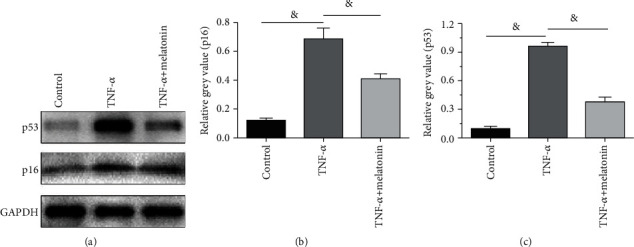
Analysis of protein expression of p16 and p53. Protein expression of p16 and p53 in annulus fibrosus (AF) cells was analyzed by western blot assay. (a) Protein bands of GAPDH, p16, and p53. (b, c) Densitometry analysis of p16 and p53. All data are expressed as mean ± SD (*n* = 3). ^&^Indicates a statistical difference (*p* < 0.05).

Results showed that protein expression of both p16 and p53 in the TNF-*α* group was obviously elevated compared with the control cells, whereas melatonin partly declined their protein expression in the TNF-*α* group (Figure 5).

### 3.6. Activity of the NF-*κ*B Pathway

**Figure 6:**
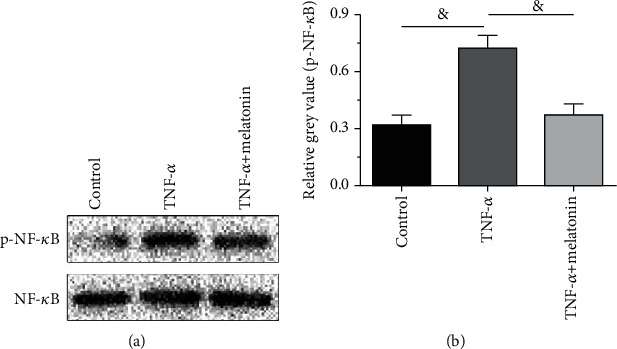
Analysis of activation of the NF-*κ*B pathway. Activation of the NF-*κ*B pathway was evaluated by the protein expression of phospho-NF-*κ*B. The numeric data are expressed as mean ± SD (*n* = 3). ^&^Indicates a statistical difference (*p* < 0.05).

To further study the role of oxidative stress reaction in this pathological process, we analyzed the activity of the downstream NF-*κ*B. Results showed that protein expression of phosphor NF-*κ*B in the TNF-*α* group was significantly increased, whereas melatonin partly decreased its protein expression in the TNF-*α* group (Figure 6).

## 4. Discussion

Intervertebral disc degeneration is a main reason of spine degenerative diseases [1]. It has been found that inflammation response is significantly promoted within the degenerative disc tissues, which indicated that inflammation response is involved in the progression of disc degeneration [2, 20]. In fact, several studies have reported that inflammatory cytokines (i.e., TNF-*α* and IL-1*β*) are identified to be significantly increased within the degenerative disc tissue. Moreover, previous studies have demonstrated that inflammatory cytokines often induce degeneration-like changes of disc cells, such as cell apoptosis, cell senescence, aggravation of matrix catabolism, and attenuation of matrix anabolism [3, 8, 14, 26, 29]. As an important structure of the intervertebral disc, inhibition of inflammation response and the secondary pathological changes of disc AF tissue may be a promising strategy to retard disc degeneration.

Cell senescence is an important cellular feature during disc degeneration [11, 12]. Several previous studies have identified senescent cells within the degenerative human disc tissue and demonstrated a positive relationship between cell senescence and progression of disc degeneration [30–32]. In fact, lots of basic studies have proven that cell senescence participated in lots of pathological factor-induced degenerative alterations in disc cells, such as mechanical compression, inflammatory cytokines, acidic pH, and high glucose niche [4, 8, 9, 13, 26, 33–35]. Based on the above findings, it has been established that inhibition of cell senescence may be helpful to retard the progression of disc degeneration.

In this study, we evaluated AF cell senescence through some common parameters, such as cell proliferation potency, SA-*β*-Gal staining, telomerase activity, and protein expression of senescence markers (p16 and p53). Our results found that TNF-*α* obviously declined cell proliferation potency and telomerase activity, increased SA-*β*-Gal activity, and upregulated protein expression of senescence markers (p16 and p53) compared with the control cells, indicating that inflammatory cytokine TNF-*α* promotes disc AF cell senescence. In line with us, several previous studies also showed that inflammatory cytokines induce disc cell senescence in vitro [8, 14, 20, 26]. However, an intact disc organ consists of three parts: the peripheral AF tissue, the central nucleus pulposus tissue, and the upper and lower cartilage endplates. According to our cell isolation method, some other cells from adjacent disc tissue may exist in the isolated cell pellets, which may bring inference to the actual results to some extent. If some specific cell markers of AF cells are identified in the future, the researchers can further purify the isolated AF cells, and thus, this limitation can be resolved better.

Melatonin (N-acetyl-5-methoxytraptamine) is an indolyl hormone released from the pineal gland, which has protective effects on the nervous system and other organs [24, 25]. A previous study of Turgut et al. demonstrated that there is a close negative relationship between serum melatonin levels in patients with intervertebral disc herniation, and the serum levels of melatonin might be affected by the disc degeneration process [36]. Additionally, several recent studies have demonstrated that melatonin protects against disc degeneration [28, 37]. In the present study, we also found that melatonin attenuated the effects of TNF-*α* on AF cell senescence, reflected by the increased cell proliferation potency and telomerase activity and decreased SA-*β*-Gal activity and protein expression of senescence markers (p16 and p53) of AF cells in the TNF-*α*+melatonin group. Our findings also suggest that melatonin can inhibit degenerative changes of disc AF cells through protection against cellular senescence.

Oxidative stress is involved in the pathogenesis of many age-related diseases including intervertebral disc degeneration [38]. Oxidative stress is associated with excessive production of intracellular ROS [39]. Moreover, the NF-*κ*B pathway is often activated in the process of oxidative stress [39]. In this study, we found that inflammatory cytokine TNF-*α* increased intracellular ROS content and activated the NF-*κ*B pathway. Our results are consistent with a previous study that the ROS/NF-*κ*B pathway participates in the regulation of inflammatory cytokines on disc cell behaviors [26]. However, when melatonin was added into the culture medium of AF cells in the TNF-*α* group, ROS content and activity of the NF-*κ*B pathway of AF cells were decreased. These results suggest that melatonin may inhibit the regulation of TNF-*α* on AF cell senescence through attenuating oxidative stress.

## 5. Conclusion

In this study, we investigated the effects of melatonin on AF cell senescence in an inflammation environment and the role of the ROS/NF-*κ*B pathway in this process. Our results demonstrated that melatonin can attenuate AF cell senescence through suppressing the ROS/NF-*κ*B pathway in an inflammation environment. This study sheds a new light on the protective effects of melatonin against AF cell senescence and indirectly provides that melatonin may be promising to inhibiting excessive inflammation response to retard the progression of disc degeneration.

## Data Availability

All data used to support the findings of this study are included within the article.
